# Use of flow cytometry and total viable count to determine the effects of orange juice composition on the physiology of *Escherichia coli*

**DOI:** 10.1002/fsn3.756

**Published:** 2018-08-13

**Authors:** Amir H. P. Anvarian, Madeleine P. Smith, Tim W. Overton

**Affiliations:** ^1^ Bioengineering School of Chemical Engineering The University of Birmingham Birmingham UK; ^2^ Institute of Microbiology & Infection The University of Birmingham Birmingham UK; ^3^ Present address: National Centre for Food Manufacturing Holbeach Technology Park University of Lincoln Holbeach Lincolnshire UK

**Keywords:** amino acids, *E. coli*, flow cytometry, orange juice, viable but nonculturable

## Abstract

Orange juice (OJ) contains numerous compounds some of which are known to play key roles in growth and survival of bacteria. This study aimed to investigate the effects of natural or processing‐induced variations in OJ composition on the physiology of *Escherichia coli*. OJ and model OJ (MOJ) samples containing various sugars, organic acids, amino acids, or ascorbic acid were inoculated with *E. coli* K‐12 MG1655 in different growth phases. The culturability, viability, and physiology of the cells were investigated during storage using plate counting and flow cytometry. Generally, stationary‐phase cells displayed the greatest survival in both MOJ and OJ. Increase in incubation temperature from 4 to 22.5ºC caused a significant decrease in both healthy and culturable cell populations. Supplementation of MOJ with ascorbic acid and amino acids increased both the viability and culturability of the cells. Similar trends were observed in amino acid‐supplemented OJ, albeit at a slower rate. In contrast, variations in sugar or organic acid composition had negligible effects on the physiological status of the cells. In summary, natural variation in ascorbic acid or amino acid concentrations could potentially have an adverse effect on the microbiological safety of orange juice.

## 1 INTRODUCTION

Minimally processed foods are currently popular both with consumers and manufacturers. They are perceived as being “natural,” “healthy,” and more nutritious than extensively processed foods (Ragaert, Verbeke, Devlieghere, & Debevere, 2004). A popular minimally processed food is orange juice (OJ). OJ is available to consumers in a variety of formats, from the more heavily processed (e.g., Ultra Heat Treated, ambient stable) to minimally processed (freshly squeezed). One potential drawback of minimally processed foods is increased microbiological risk, as food processing techniques are frequently used primarily to reduce numbers of viable microbes in foods, both for prevention of spoilage and removal of foodborne pathogens. This results in a shorter shelf life, requirement for cold chain storage and potential for foodborne illness.

Orange juice (OJ) contains a wide range of compounds, mainly sugars (sucrose, glucose, and fructose) and organic acids (citric and malic acids) as well as minerals (chiefly potassium), various amino acids, and ascorbic acid. Some of these compounds have been shown to play an important role in growth and/or survival of *E. coli*. For instance, glucose and fructose could be utilized by *E. coli* as a carbon source for metabolism. On the other hand, citric and malic acids have been shown to exhibit antimicrobial activity against *E. coli* (Bjornsdottir, Breidt, & McFeeters, 2006; Eswaranandam, Hettiarachchy, & Johnson, 2004; Raybaudi‐Massilia, Mosqueda‐Melgar, Soliva‐Fortuny, & Martín‐Belloso, 2009). With regard to amino acids present in OJ, arginine and glutamate are the major components of the acid resistance mechanisms in *E. coli* (Richard & Foster, 2004). Moreover, it has been suggested that proline, the most abundant amino acid in OJ, could increase the survival of *E. coli* in an acidic model apple juice (Reinders, Biesterveld, & Bijker, 2001). In addition, ascorbic acid has been shown to act not only as an antioxidant but also an antimicrobial against *E. coli* particularly in combination with organic acids (Padayatty et al., 2003; Tajkarimi & Ibrahim, 2011).

Flow cytometry (FCM) is a rapid, single‐cell analysis technique that allows analysis of bacterial viability and physiology without the requirement for growth on agar plates (Comas‐Riu & Rius, 2009). Samples of bacteria suspended in a liquid (e.g., a liquid foodstuff, a fermentation broth or water) are passed in a stream in front of a Laser beam, where particles suspended in the liquid are illuminated one at a time, up to several thousand per second. Scattered light is detected using sensors in line with and perpendicular to the Laser beam, indicating particle size and “granularity” (a measure of particle optical complexity), respectively. In addition, particle fluorescence is measured; staining samples with fluorescent dyes allows determination of aspects of bacterial physiology and viability (Bridier, Hammes, Canette, Bouchez, & Briandet, 2015). The major advantages of FCM are speed of analysis (minutes); detection of subpopulations present in a sample (e.g., numbers of live, dead, and injured bacteria); and the lack of reliance on growth on agar plates, thus allowing analysis of not only culturable but also nonculturable, and thereby also VBNC (viable but nonculturable), bacteria (Nebe‐Von‐Caron, Stephens, Hewitt, Powell, & Badley, 2000).

The objective of this study was to test whether changes in concentrations of components of OJ brought about by natural variations (seasonal changes in composition or changes in cultivar) or processing‐induced changes in composition could influence the physiology of *E. coli*. A model orange juice (MOJ) solution (Table 1) was used in order to investigate the role of each component independently of the inherent variability in the composition of real fruit juice (Reinders et al., 2001) and to test known sugar or acid compositions mimicking seasonal and/or cultivar variability of OJ (Kelebek & Selli, 2011; Villamiel & Martõ, 1998). Furthermore, using a model system eliminated the need for chemical analysis of OJ composition. We tested the physiological response of *E. coli* to this simple MOJ, to OJ as a comparison, and to MOJ with altered composition with regard to organic acids, sugars, and amino acids to model variations in OJ composition. We utilized flow cytometry as a rapid technique for assessing bacterial physiology and viability in a nongrowth‐dependent manner (Anvarian, Smith, & Overton, 2016). Finally, as altering the amino acid content of MOJ caused a change in *E. coli* physiology, we determined the effect on *E. coli* of supplementing OJ with amino acids.

**Table 1:** Composition of model orange juice (MOJ) as compared to literature values of freshly squeezed orange juice composition

	MOJ (g/L)	OJ	
		Range of Literature values (g/L)	RSK^a^ (g/L) Mean [Min, Max]
Sugars	85	65.1–120.2^b^	NS
Sucrose	45	29.4–61.4^b^	33.0 [NS, 47.0]
Glucose	20	13.8–33.2^b^	28.0 [20.0, NS]
Fructose	20	17.0–34.2^b^	30.0 [22.0, NS]
Organic acids	11.5	5.7–18.1^d^	NS
Citric acid	9.5	5.1–14.3^d^	9.4 [7.6, 11.5]
Malic acid	2	0.4–4.0^d^	1.7 [1.1, 02.9]
Buffering agent			
Potassium citrate	5.02		
Potassium		1.2–3.0^c^	1.9 [1.4, 2.3]
pH	3.23 ± 0.01	3.25 ± 0.05^e^	
Osmolality (mOmsol/kg)	488 ± 15	497 ± 2^e^	

*Notes*

NS: not specified. Compositional data are taken from a variety of sources: ^a^From RSK (1987); Internationally accepted standard for unadulterated freshly squeezed OJ.^b^From Niu et al. (2008), Kelebek, Selli, Canbas, and Cabaroglu (2009) Kelebek and Selli (2011) and Villamiel and Martõ (1998).^c^From Robards and Antolovich (1995).^d^From Capilla, Navarro, Sendra, and Izquierdo (1988), Niu et al. (2008), Kelebek et al. (2009) and Kelebek and Selli (2011).^e^Measured in this study for freshly squeezed OJ, filtered through 1.2‐μm filter, mean ± standard deviation of 3 samples.

## 2 MATERIALS AND METHODS

### 2.1 Microbiological methods

*E. coli* K‐12 strain MG1655 was used due to its suitability as a surrogate strain for *E. coli* O157:H7 in food microbiological studies (Anvarian et al., 2016; Valdramidis, Geeraerd, & Van Impe, 2007). A single colony of *E. coli* was taken from a nutrient agar (Oxoid) plate inoculated into 20 ml of 2 × LB (Luria‐Bertani broth; 20 g/L tryptone, 10 g/L yeast extract, 10 g/L NaCl) in a 250‐mL conical flask and was grown for 18 hr at 37°C and 150 rpm shaking. The overnight culture was subsequently diluted (1:1,000) into 50 ml of fresh 2 × LB medium in a 250‐ml conical flask and grown under the same conditions until the desired OD_650_ was reached. Cultures at an OD_650_ of 0.5 were used for experiments with supplementation of MOJ and OJ with organic acids, amino acids, and ascorbic acid. Cells were harvested by centrifugation (10 min at 3,256 *g*), washed, and dispersed in PBS (phosphate‐buffered saline; 8.0 g/L NaCl, 0.2 g/L KCl, 1.15 g/L Na_2_HPO_4_, and 0.2 g/L KH_2_PO_4_, pH 7.3). Then, 50 μl of cell suspension containing 3 × 10^9^ *E. coli* cells was added to 15 ml of either MOJ or OJ. Samples were taken for analysis immediately prior to addition to OJ or MOJ, immediately after addition, and after specified time intervals.

### 2.2 Orange juice and model orange juice

Freshly squeezed OJ was obtained from a local retailer. The total viable count (TVC) of the freshly squeezed OJ was <10 CFU/g. Freshly squeezed OJ was centrifuged at 17,696 *g* for 40 min to remove pulp. The supernatant (pulp‐free OJ) was filtered through sterile 1.2‐μm filter paper to permit analysis by flow cytometry. A model orange juice (MOJ) was developed containing the major components of OJ (Table 1). The constituents of MOJ were dissolved in deionized water and filter sterilized using a 0.22‐μm filter (Millipore).

### 2.3 Analysis of bacterial viability and physiology

Viability was determined using TVC; bacteria were diluted in maximum recovery diluent (8.5 g/L NaCl, 1 g/L peptone) and plated on nutrient agar medium. Plates were incubated at 37°C for 48 hr before counting. Flow cytometry was used as an alternative technique for measuring bacterial physiology. A BD Accuri C6 (BD, Oxford, UK) flow cytometer was used. Samples were diluted in PBS and stained with propidium iodide (PI; Sigma) and Bis‐(1,3‐dibutylbarbituric acid) trimethine oxonol (DiBAC_4_(3) or BOX; Life Technologies) to determine viability and membrane potential, respectively (Anvarian et al., 2016). PI is a red nucleic acid dye that can only enter bacteria with a compromised inner membrane, signifying death. BOX is a green lipophillic dye that can only enter bacteria with no membrane potential, signifying either injured or dead cells. A 200‐μg/ml stock solution of PI was made up in distilled water and added to samples at a final concentration of 4 μg/mL. A 10 mg/mL stock solution of BOX was made up in dimethyl sulphoxide (DMSO) and added to samples at a final concentration of 4 μg/mL, along with 400 μM EDTA. Samples were excited by a 488 nm laser. Cells were gated from particulate noise using a forward scatter height threshold. 20,000 data points were collected per sample, at a maximum of 2,500 events/s. Fluorescence was detected using channels FL1 (533/30 BP filter; BOX) and FL3 (670 LP filter; PI). Data were analyzed using CFlow (BD). Live cells and heat‐killed cells were used as controls to set up gating.

## 3 RESULTS AND DISCUSSION

### 3.1 Effect of growth phase and incubation temperature on E. coli physiology in orange juice and model orange juice

The physiology and culturability of *E. coli* in different growth phases, from early log phase to late stationary phase, was compared over time in orange juice at 4°C (indicative of refrigerated storage) or 22.5°C (to model ambient storage). In order to compare total viable count data with viability and physiology data generated by flow cytometry, orange juice was filtered, as the cloud of OJ interferes with bacterial detection using FCM. The effects of filtering OJ with filters of different pore sizes on *E. coli* are previously described (Anvarian et al., 2016). In addition, a model orange juice (MOJ) was developed to allow modelling of OJ composition. Previous studies have mainly used MOJ to study browning (e.g., (Shinoda, Komura, Homma, & Murata, 2005)) but not effects on bacterial viability or physiology. The composition of MOJ mimics the composition of OJ found in the literature (Table 1). The MOJ used in this study initially consisted of only the main sugars, organic acids, and minerals of OJ (Robards & Antolovich, 1995).

**Figure 1:**
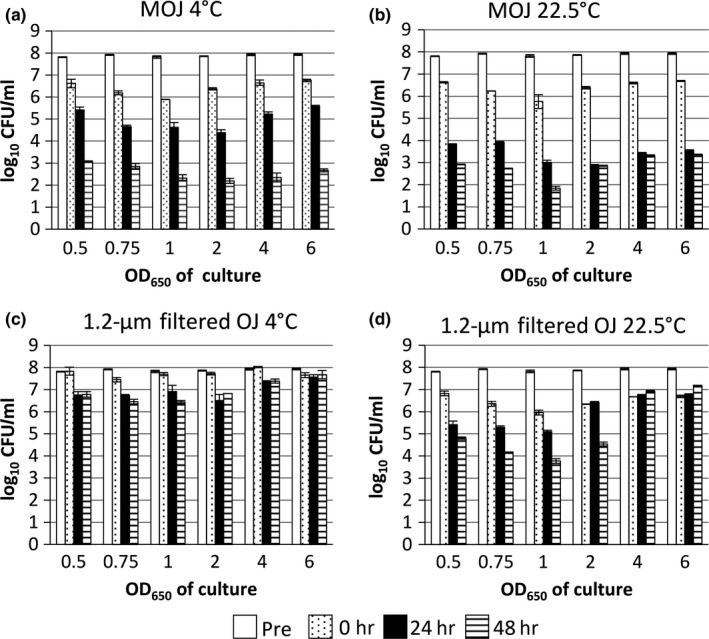
The effects of cell growth phase and incubation temperature on the TVC of *E. coli* in 1.2‐μm filtered orange juice (OJ) and model orange juice (MOJ). TVC was determined before addition to OJ or MOJ (pre), immediately after addition (0 hr), and after 24 and 48 hr. Mean TVC ± *SD* is shown for two independent cultures

Three general trends were observed in the TVC data (Figure 1). First, incubation in OJ at 22.5°C (Figure 1d) resulted in greater loss of culturability than incubation in OJ at 4°C (Figure 1c). This has been observed before in growth media and foodstuffs (Garcia‐Graells, Hauben, & Michiels, 1998; Uljas & Ingham, 1998) and can be attributed to a combination of the increased metabolic activity of bacteria, increased permeability of the cell membrane to protons and increased dissociation of weak acids at higher temperatures (Álvarez‐Ordóñez, Valdés, Bernardo, Prieto, & López, 2013). This change in culturability depending upon incubation temperature was not observed to a great extent in MOJ, although a similar trend can be observed in samples incubated in MOJ for 1 day (Figure 1a,b).

Second, comparing culturability in 1.2‐μm filtered OJ and MOJ (Figure 1 panels c & d versus a & b), loss of culturability was far greater in MOJ, despite the two fluids having similar pH, osmolarity, and major chemical components (Table 1). This is indicative of other components of OJ having a protective effect on *E. coli*. Even when passed through a 1.2‐μm filter, OJ contains many compounds that might have a protective effect on *E. coli* (Anvarian et al., 2016). This phenomenon has been observed before in other studies comparing foodstuffs and model foodstuffs (Nualkaekul & Charalampopoulos, 2011; Uljas & Ingham, 1998).

**Figure 2:**
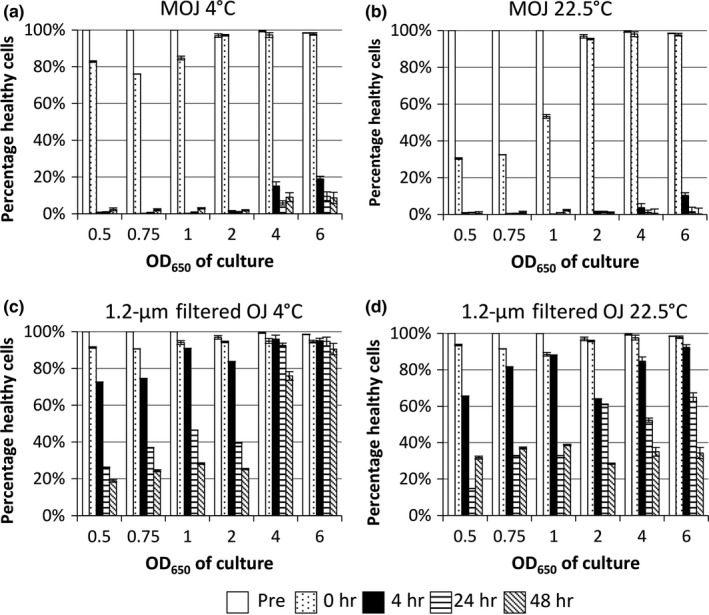
The effects of cell growth phase and incubation temperature on the viability of *E. coli* in 1.2‐μm filtered orange juice (OJ) and model orange juice (MOJ). Cell viability and physiology were determined by FCM before addition to OJ or MOJ (Pre), immediately after addition (0 hr), and after 4, 24, and 48 hr. Mean percentage healthy cells (PI^−^ BOX^−^) ± *SD* are shown for two independent cultures

Finally, stationary phase *E. coli* tended to be more resistant to losses in culturability than exponential phase bacteria. This trend was most marked in OJ samples incubated for 48 hr at 22.5°C (Figure 1d). This is a widely reported phenomenon (Arnold & Kaspar, 1995; Benjamin & Datta, 1995; Chung, Bang, & Drake, 2006), thought to be due to a combination of lower growth rate and induction of stress responsive and detoxification pathways in stationary phase. As well as TVC analysis, samples incubated in 1.2‐μm filtered OJ and MOJ were analyzed using flow cytometry. Two stains were used at the same time: propidium iodide (PI), a red dye which can only enter dead cells; and bis‐oxonol (bis‐(1,3‐dibutylbarbituric acid) trimethine oxonol; BOX), a green lipophilic stain that only enters cells with a collapsed membrane potential and thus stains cells that are not actively respiring (injured or dead cells). Thereby FCM can be used to rapidly and directly measure aspects of cell viability and physiology, without the need for culture. Three staining combinations are possible: healthy cells, actively respiring with a membrane potential (PI^−^ BOX^−^); “injured” cells, viable but without a membrane potential (PI^−^ BOX^+^); and dead cells (PI^+^ BOX^+^). FCM data are represented in two ways: percentage of healthy cells (defined as BOX^−^ PI^−^ bacteria, thereby alive and with a membrane potential; Figure 2), and numbers of healthy cells, permitting comparison with TVC data (Supporting information Figure S1).

Upon addition to MOJ or OJ, the percentage of healthy cells decreased in most samples (0 hr sample); this decrease was larger in MOJ than OJ (Figure 2 panels a & b vs. c & d). In MOJ, log phase *E. coli* showed a greater decrease in healthy percentage, especially at 22.5°C (Figure 1b). As with TVC data, there were general trends that the percentage of healthy cells decreased more in MOJ compared to OJ; at 22.5°C compared to 4°C; and for log phase compared to stationary phase cells.

Comparison of FCM healthy cell numbers (Supporting information Figure S1) with TVC data (Figure 1) reveals that, for all conditions, healthy cell numbers as determined by FCM (PI^−^ BOX^−^ cells) were higher than TVC. The discrepancy is caused by cells with the VBNC (viable but nonculturable) phenotype (Li, Mendis, Trigui, Oliver, & Faucher, 2014), commonly encountered in bacteria experiencing stressful conditions. VBNC cells are viable and metabolically active but cannot be cultured. The exact biochemical and physiological causes of the VBNC phenotype are not completely understood, but it is believed to be a survival mechanism (Ramamurthy, Ghosh, Pazhani, & Shinoda, 2014). It should be noted that the VBNC phenotype observed is specific to the culture conditions used here, under which the bacteria were not culturable; use of different media might permit recovery of culturability. Nonetheless, this highlights the utility of flow cytometry as a technique for assessing viability in a non growth‐dependent manner.

### 3.2 Effects of altering the sugar and organic acid composition of model orange juice

As the concentrations of sugars present in OJ can fluctuate according to season, variety of orange, and other factors, the effect of changes in MOJ sugar concentrations (Supporting information Table S1) on the physiology of mid‐exponential phase *E. coli* at 4°C was tested. The sugar concentration of these MOJ compositions was within the range of sugar concentrations naturally found in OJ (Table 1). The pH of the MOJ solutions with altered sugar concentrations altered very little, but the osmolarities were dramatically different due to the change in sugar concentration. As before, the number of culturable bacteria as measured using TVC analysis was lower than viable or healthy bacteria as determined using FCM (defined as those not staining with PI or those staining with neither PI nor BOX, respectively). However, no difference was observed between viability as assessed by FCM or culturability of *E. coli* in MOJ samples containing different sugar concentrations (Supporting information Figure S2a).

This experiment was repeated with alteration of organic acid content in MOJ; the concentrations of citric and malic acids in OJ can vary widely depending on the harvest season, origin or the variety/cultivar of the orange fruit as of well as the OJ extraction techniques used. MOJ solutions containing different concentrations of citric and malic acids were prepared (Supporting information Table S1). The pH of the MOJ solutions was kept relatively constant (pH 3.19–3.23) by changing the concentration of potassium citrate. Nevertheless, in order to simulate the citrate buffer mixture of real OJ, the molar ratio of citric acid to potassium citrate (2.63–3.13) was kept as close to 3:1 (previously reported by (Lanford, 1942)) as possible. The concentration of potassium in MOJ solutions (0.97–3.25 g/L) and the molar ratio of citric acid to malic acid (2.63–3.13) were close to the range reported for OJ (Robards & Antolovich, 1995; Saccani et al., 1995). With the exception of the composition of MOJ solutions, the method used for this experiment was identical to that described above for sugar experiments (Supporting information Figure S2b).

While there were no significant differences between viability or culturability of bacteria incubated in medium and low acid MOJ, or medium and high acid MOJ, there was a significant difference in the numbers of healthy bacteria as determined by FCM and culturable bacteria (as determined by TVC) between low and high acid MOJ. As such, increasing organic acid concentration decreased viability and culturability. The biochemical and physiological reason for this is presently unknown, but as altering organic acid concentrations did not significantly change the pH of MOJ, the organic acid concentrations themselves are probably the cause of loss of viability and culturability.

### 3.3 Effects of altering the ascorbic acid content of model orange juice

Ascorbic acid (vitamin C) is a natural component of OJ and is desired for its health benefits, including its antioxidant properties. In order to study the effects of ascorbic acid on the viability and culturability of exponential‐phase *E. coli*, MOJ was supplemented with different concentrations of L‐ascorbic acid: 0.5 g/L, chosen to mimic the typical concentration in OJ; and 1, 5, or 10 g/L, to model commercial OJ brands supplemented with high concentrations of ascorbic acid (>7 g/L). The pH and osmolality of MOJ with different concentrations of ascorbic acid are shown in Supporting information Table S2.

**Figure 3:**
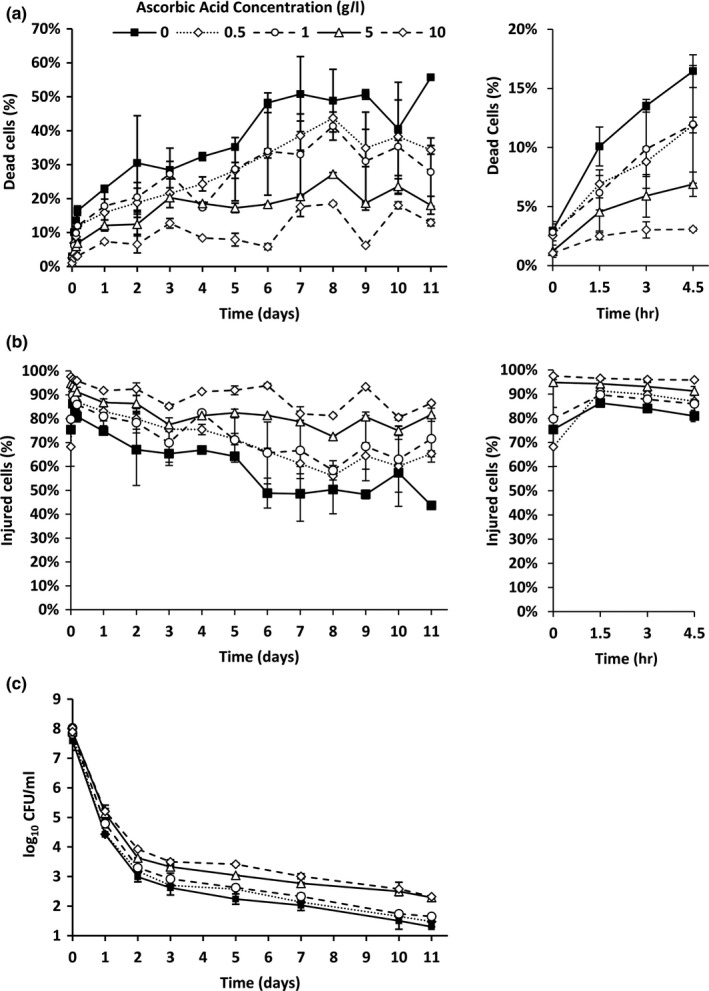
The effects of ascorbic acid content of MOJ on the viability of exponential phase *E. coli*. (a) Percentage of dead (PI^+^) cells measured by FCM. (b) Percentage of injured (PI^−^ BOX^+^) cells measured by FCM. (c) TVC. Panels on the right show magnified view of 0–4.5 hr postaddition. Error bars are the ± SD of the mean values (*n *=* *2)

Analysis using FCM revealed that increasing the ascorbic acid concentration decreased the percentage of dead (PI^+^) bacteria in MOJ at all timepoints (Figure 3a). Compared to un‐supplemented MOJ, addition of 0.5 g/L ascorbic acid caused a significant reduction in the dead population. Numbers of dead bacteria were not significantly different in MOJ with 0, 0.5% or 1% ascorbic acid, but were significantly different in MOJ with 5% or 10% ascorbic acid. The decrease in the proportion of dead bacteria observed at increased concentrations of ascorbic acid did not translate to an increase in healthy (PI^−^ BOX^−^) bacteria; instead, the percentage of injured bacteria without a membrane potential (PI^−^ BOX^+^) increased with increasing ascorbic acid concentration (Figure 3b).

Culturability of bacteria as measured by TVC was significantly higher in MOJ supplemented with 5 or 10 g/L ascorbic acid (Figure 3c). Culturability was not significantly different in MOJ with zero, 0.5 g/L or 1 g/L ascorbic acid, indicating that protective effects are only visible at ascorbic acid concentrations significantly greater than those naturally found in OJ under these conditions.

Ascorbic acid has been shown to have both positive and negative effects on microbial viability; effects are dependent upon the physicochemical properties of the food matrix, in particular the presence of organic acids, and the bacterial species. Ascorbic acid is known to increase survival of probiotic bacteria (Shah, Ding, Fallourd, & Leyer, 2010) and has been shown to induce catalase expression in *E. coli*, potentially increasing resistance to oxidative stress (Richter & Loewen, 1981). Indeed, it is known that supplementation of agar plates with catalase can restore culturability of VBNC *E. coli* (Mizunoe, Wai, Takade, & Yoshida, 1999). The protective effect of ascorbic acid observed here may reflect induction of catalase and thus an increased resistance to oxidative stress; this should be taken into account when devising microbiological safety strategies for ascorbic acid supplemented OJ products.

### 3.4 Effect of altering the amino acid concentration of MOJ

**Figure 4:**
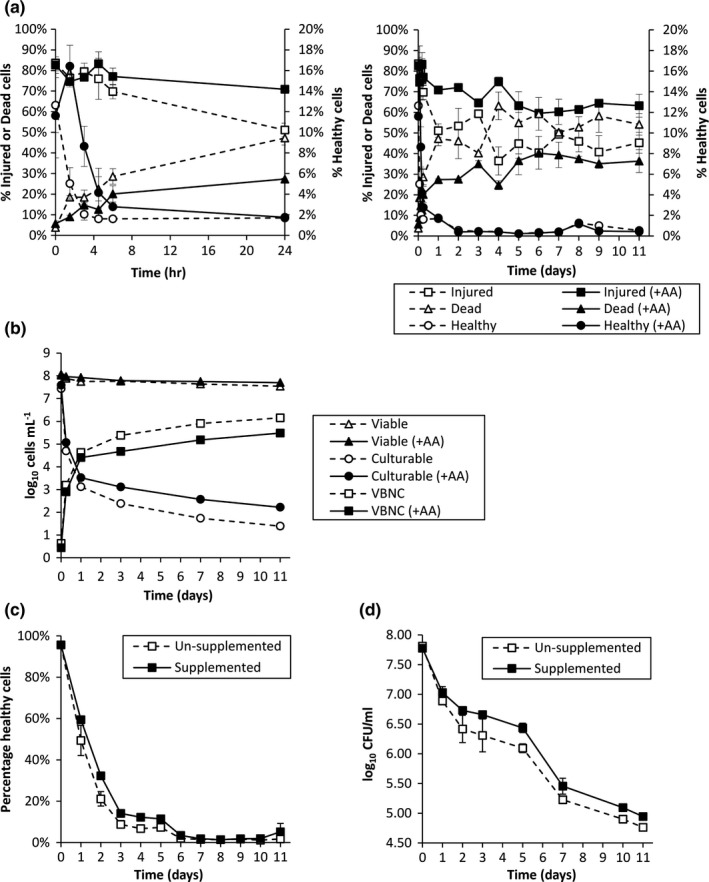
The effects of amino acid supplementation of MOJ (a, b) or OJ (c, d) on the viability of exponential phase *E. coli*. (a) Percentage of healthy (PI^−^ BOX^−^), injured (PI^−^ BOX^+^), and dead (PI^+^) bacteria in MOJ with or without amino acid supplementation measured using FCM. Panel on left is magnified view of 0–24 hr postaddition. (b) Comparison of numbers of viable (PI^−^) bacteria in MOJ with or without amino acid supplementation determined by FCM and culturable bacteria determined by TVC. VBNC numbers calculated by viable minus culturable. Error bars are the standard deviation of mean values. (c) Percentage healthy (PI^−^ BOX^−^) bacteria and (d) TVC in OJ with or without amino acid supplementation. Error bars are the ± *SD* of the mean values, *n* = 2

The effect of addition of amino acids on the culturability and physiology of exponential‐phase *E. coli* in MOJ at 4°C was determined (Figure 4). MOJ was supplemented with the eight most abundant amino acids found in OJ at concentrations similar to those found in OJ (Supporting information Table S3).

Supplementation of MOJ with amino acids resulted in a significantly greater percentage of viable bacteria as determined by FCM (Figure 4a). At 1.5 hr postinoculation, amino acid supplementation increased the percentage of healthy bacteria (PI^−^ BOX^−^) threefold. From 4.5 hr postinoculation, no significant difference was observed between the percentage of healthy bacteria in MOJ with or without amino acids. Nevertheless, a greater percentage of injured cells (PI^−^ BOX^+^) and a lower percentage of dead cells (PI^+^ BOX^+^) were observed in MOJ samples supplemented with amino acids. Amino acid addition also led to an increase in the culturability of *E. coli* (Figure 4b). Numbers of VBNC cells were also lower in MOJ containing amino acids.

### 3.5 Effect of amino acids supplementation of OJ

Considering the apparent beneficial effects of amino acids on the viability and culturability of *E. coli* in MOJ, the same mixture of amino acids was added to OJ and *E. coli* physiology was determined (Figure 4c). The rate of decrease in the size of the healthy population in OJ as determined by FCM was significantly lower compared to MOJ samples. At all timepoints, the mean percentage of healthy cells was greater in amino acid‐supplemented samples. Based on these results, it could be suggested that amino acids in OJ exert a beneficial effect on the viability of *E. coli* in OJ. This was also observed when comparing the TVC of *E. coli* incubated in OJ with and without amino acid supplementation (Figure 4d).

Proline, the major amino acid present in orange juice, is a major osmoprotectant in *E. coli*. Previous studies have shown that proline is protective for *E. coli* O157:H7 in model apple juice (Reinders et al., 2001). In addition, both glutamate and aspartate have been shown to increase acid tolerance in *E. coli* (Rowbury, Humphrey, & Goodson, 1999). It is probable that a combination of these two effects is responsible for the protective effect of amino acid supplementation observed here. These data are potentially important for the microbiological safety of food products containing amino acid‐supplemented fruit juices.

## 4 CONCLUSIONS

In summary, the results showed that compared to exponential phase cells, stationary phase cells were more resistant to MOJ and OJ. Moreover, the increase in incubation temperature of the samples from 4°C to 22.5°C led to a greater decrease in the population of healthy and/or culturable cells. The differences observed between the results obtained for MOJ and OJ samples were most likely due to the presence of minor components of the OJ not present in MOJ. Further work is needed to study the exact nature of these compounds and to identify those responsible for the observed discrepancy. Moreover, in this study, the pH of MOJ was kept relatively constant. The effects of change in pH of OJ or MOJ within the ranges naturally found in OJ on the physiological response of *E. coli* also deserve further study.

The results also showed that the change in sugar or organic acid concentration of MOJ had no or little effect on the viability and/or culturability of the cells. On the other hand, changes in the concentrations of ascorbic acid and amino acids affected the viability and/or physiology of *E. coli* in MOJ. It would be interesting to utilize this information for designing a predictive model in order to predict the physiological response of *E. coli* in OJ. This could lead to a better understanding of the microbiological safety of supplemented fruit juices.

Other studies have shown that while some VBNC *E. coli* are capable of resuscitation and regaining their culturability, some are not, and the resuscitation depends on the various environmental and chemical stimuli (Arana et al., 2007; Pinto, Almeida, Almeida Santos, & Chambel, 2011; Wesche, Gurtler, Marks, & Ryser, 2009). Although the results of the current study showed that inoculation of *E. coli* in MOJ or OJ could induce the VBNC state, the possibility of resuscitating these cells was not investigated. FCM data could be used as a suitable guide for developing appropriate recovery and resuscitation media for OJ‐induced VBNC cells. This work also highlights the utility of FCM as a rapid analytical technique that does not rely upon growth.

## CONFLICT OF INTEREST

TWO and AA were paid speakers expenses by BD for speaking at BD Accuri users' events.

## ETHICAL STATEMENTS

This study does not involve any human or animal testing.

Informed consent: Not applicable.
