# Supplementary material for: Use of flow cytometry and total viable count to determine the effects of orange juice composition on the physiology of Escherichia coli
Source: Food Sci Nutr. 2018 Aug 13;6(7):1817–25. doi: 10.1002/fsn3.756 (PMC6189610; doi:10.1002/fsn3.756)
Supplement: Supplementary file 1 [file FSN3-6-1817-s001.pdf]

Supplemental information

Use of flow cytometry and total viable count to determine the effects of orange juice composition on the physiology of *Escherichia coli*.

Amir HP Anvarian[1†], Madeleine P Smith[1], Tim W Overton[1,2]*

[1] Bioengineering, School of Chemical Engineering, and [2] Institute of Microbiology & Infection, The University of Birmingham, Birmingham B15 2TT, UK

* Corresponding author: t.w.overton@bham.ac.uk; Telephone: +44 121 4145306; ORCID 0000-0003-3050-2549

Supplemental Table S1. MOJ compositions with altered sugar or organic acid concentrations.

	Low Sugar (g·L^{-1})	Medium Sugar (g·L^{-1})	High Sugar (g·L^{-1})	Low Acid (g·L^{-1})	Medium Acid (g·L^{-1})	High Acid (g·L^{-1})
**Sugars**	**60**	**85**	**120**	**85**	**85**	**85**
Sucrose	30	45	60	45	45	45
Glucose	15	20	30	20	20	20
Fructose	15	20	30	20	20	20
**Organic Acids**	**11.5**	**11.5**	**11.5**	**6**	**11.5**	**18**
Citric Acid	9.5	9.5	9.5	5	9.5	14
Malic Acid	2	2	2	1	2	4
**Buffering Agent**						
Potassium Citrate	5.02	5.02	5.02	2.55	5.02	8.50
pH[a]	3.21 ± 0.02	3.23 ± 0.01	3.20 ± 0.03	3.19 ± 0.02	3.23 ± 0.01	3.20 ± 0.03
Osmolality (mOmsol·kg^{-1})[b]	423 ± 18	488 ± 15	640 ± 15	426 ± 11	488 ± 15	594 ± 6
Potassium content (g·L^{-1})	ND	ND	ND	0.97	1.92	3.25
**Molar Ratios**						
Citric Acid/potassium citrate	ND	ND	ND	3.13	3.02	2.63
Citric Acid/Malic Acid	ND	ND	ND	3.49	3.32	2.44

a - Mean ± standard deviation of 3 samples
b - Mean ± standard deviation of 3 samples (10 measurements per sample)
ND: Not determined

Supplemental table S2. Measured pH and osmolarities of MOJ supplemented with different concentrations of ascorbic acid

Ascorbic acid concentration ($g \cdot L^{-1}$)	pH[a]	Osmolality ($mOmsol \cdot kg^{-1}$)[b]
0	3.23 ± 0.01	488 ± 15
0.5	3.24 ± 0.02	499 ± 21
1	3.22 ± 0.03	508 ± 16
5	3.20 ± 0.03	528 ± 18
10	3.18 ± 0.02	550 ± 19

a : Mean ± standard deviation of 3 samples

b : Mean ± standard deviation of 3 samples (10 measurements per sample)

Supplemental Table S3. Composition of MOJ supplemented with amino acids

	MOJ (Amino Acid-Supplemented) Concentrations in $g\cdot L^{-1}$
Sugars	85
Sucrose	45
Glucose	20
Fructose	20
Organic Acids	11.5
Citric Acid	9.5
Malic Acid	2
Potassium Citrate	5.02
Amino Acids	2.455
L-Proline	0.794
L-Arginine	0.564
L-Aspartic Acid	0.274
L-Asparagine	0.305
L-Glutamic Acid	0.145
L-Serine	0.128
L-Alanine	0.108
γ-Aminobutyric Acid	0.237
pH[a]	3.20 ± 0.04
Osmolality (Omsol$\cdot kg^{-1}$)[b]	513 ± 21

a - Mean ± standard deviation of 3 samples

b - Mean ± standard deviation of 3 samples (10 measurements per sample)

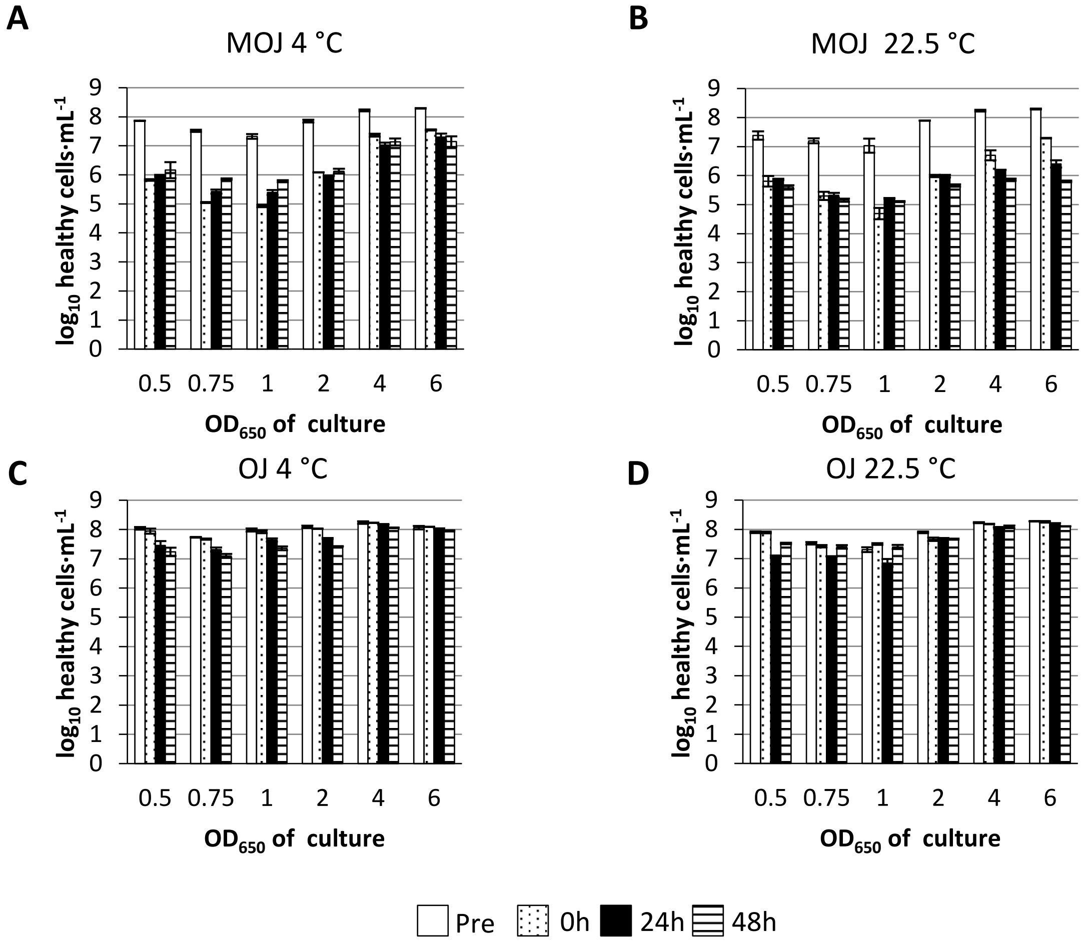

Supplemental Figure S1. The effects of cell growth phase and incubation temperature on the viability of *E. coli* K-12 in 1.2 µm-filtered orange juice (OJ) and model orange juice (MOJ). *E. coli* K-12 cultures were grown to an OD_{650} of 0.5, 0.75, 1, 2, 4 or 6 and added to MOJ or 1.2 µm-filtered OJ; samples were incubated at 4 °C or 22.5 °C. Cell viability and physiology was determined using FCM before addition to OJ or MOJ (pre), immediately after addition, (0h) and at 4, 24 and 48 hours post-addition. Mean number of healthy cells (PI^- BOX^-) ± SD is shown.

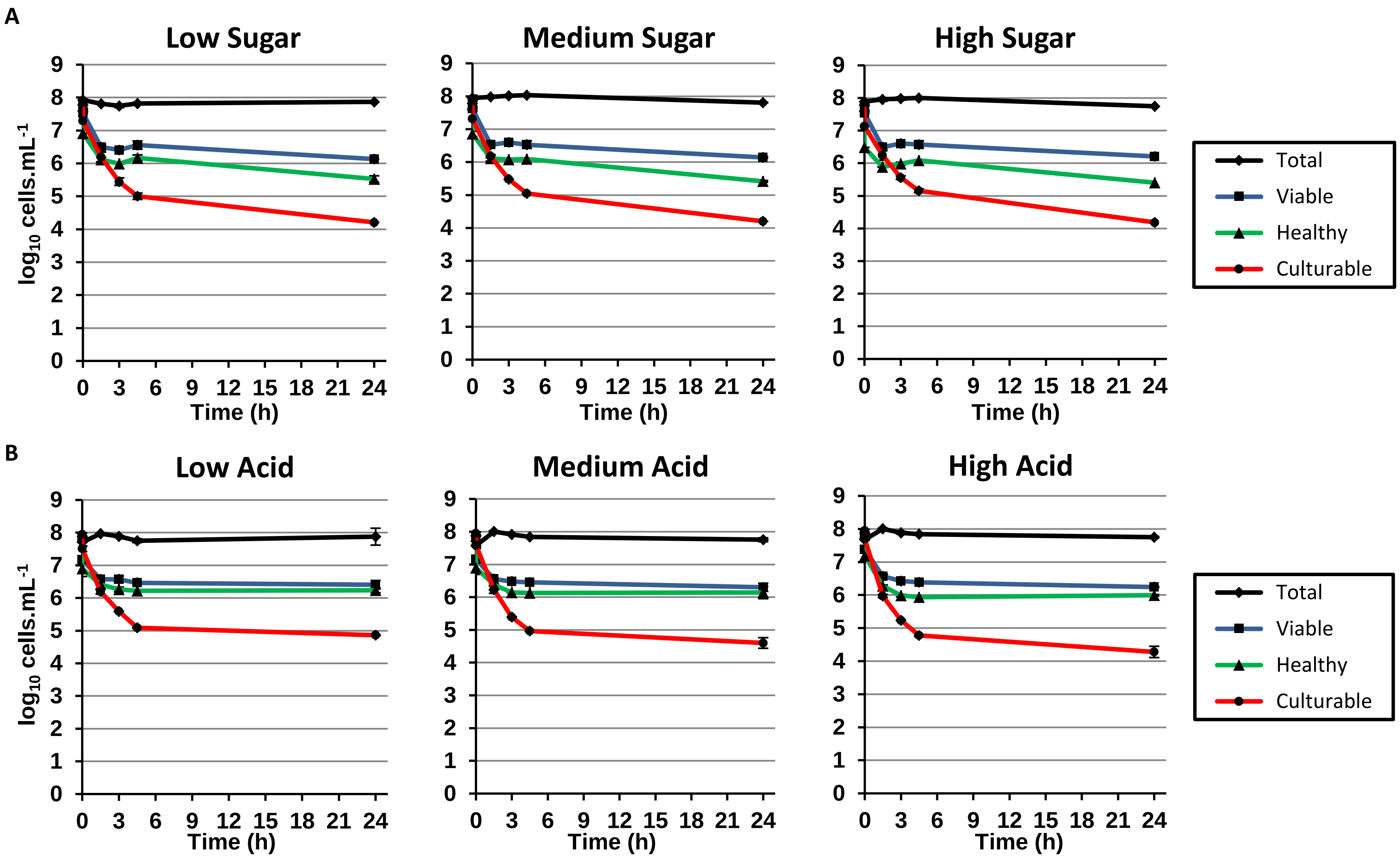

Supplemental Figure S2. The effects of changes in (A) sugar or (B) organic acid content of MOJ on the viability and culturability of exponential phase *E. coli* K-12 MG1655 (OD_{650nm} 0.5) incubated at 4 °C for 24 h. The method for this experiment was similar to that described in Figures 1 & 2, except that exponential phase *E. coli* was inoculated in MOJ solutions containing low, medium or high sugar contents (Supplemental Table S1). Culturable cells were determined using TVC, Total, Viable (PI^-) and Healthy (PI^- BOX^-) cells were determined by FCM. Data shown are mean ± SD.
